# Signal intensity and volume of carotid intraplaque hemorrhage on magnetic resonance imaging and the risk of ipsilateral cerebrovascular events: The Plaque At RISK (PARISK) study

**DOI:** 10.1016/j.jocmr.2024.101049

**Published:** 2024-06-13

**Authors:** Kelly P.H. Nies, Mueez Aizaz, Dianne H.K. van Dam-Nolen, Timothy C.D. Goring, Tobien A.H.C.M.L. Schreuder, Narender P. van Orshoven, Alida A. Postma, Daniel Bos, Jeroen Hendrikse, Paul Nederkoorn, Rob van der Geest, Robert J. van Oostenbrugge, Werner H. Mess, M. Eline Kooi

**Affiliations:** a Cardiovascular Research Institute Maastricht (CARIM), Maastricht University, Maastricht, the Netherlands; b Department of Radiology and Nuclear Medicine, Maastricht University Medical Center, Maastricht, the Netherlands; c Department of Radiology and Nuclear Medicine, Erasmus MC, University Medical Center Rotterdam, Rotterdam, the Netherlands; d Department of Neurology, Erasmus University Medical Center Rotterdam, Rotterdam, the Netherlands; e Department of Neurology, Amsterdam University Medical Center, Amsterdam, the Netherlands; f Department of Neurology, Zuyderland Medical Center, Heerlen, the Netherlands; g Department of Neurology, Zuyderland Medical Center, Sittard, the Netherlands; h School for Mental Health and Neuroscience (MHeNs) Maastricht University, Maastricht, the Netherlands; i Department of Epidemiology, Erasmus MC, University Medical Center Rotterdam, Rotterdam, the Netherlands; j Department of Radiology and Nuclear Medicine, University Medical Center Utrecht, Utrecht, the Netherlands; k Department of Radiology, Leiden University Medical Center, Leiden, the Netherlands; l Department of Neurology, Maastricht University Medical Centre, Maastricht, the Netherlands; m Department of Clinical Neurophysiology, Maastricht University Medical Centre, Maastricht, the Netherlands

**Keywords:** Atherosclerosis, Carotid artery, MRI, Intraplaque hemorrhage, Signal intensity, Stroke

## Abstract

**Background:**

The Plaque At RISK (PARISK) study demonstrated that patients with a carotid plaque with intraplaque hemorrhage (IPH) have an increased risk of recurrent ipsilateral ischemic cerebrovascular events. It was previously reported that symptomatic carotid plaques with IPH showed higher IPH signal intensity ratios (SIR) and larger IPH volumes than asymptomatic plaques. We explored whether IPH SIR and IPH volume are associated with future ipsilateral ischemic cerebrovascular events beyond the presence of IPH.

**Methods:**

Transient ischemic attack and ischemic stroke patients with mild-to-moderate carotid stenosis and an ipsilateral IPH-positive carotid plaque (n = 89) from the PARISK study were included. The clinical endpoint was a new ipsilateral ischemic cerebrovascular event during 5 years of follow-up, while the imaging-based endpoint was a new ipsilateral brain infarct on brain magnetic resonance imaging (MRI) after 2 years (n = 69). Trained observers delineated IPH, a hyperintense region compared to surrounding muscle tissue on hyper T_1_-weighted magnetic resonance images. The IPH SIR was the maximal signal intensity in the IPH region divided by the mean signal intensity of adjacent muscle tissue. The associations between IPH SIR or volume and the clinical and imaging-based endpoint were investigated using Cox proportional hazard models and logistic regression, respectively.

**Results:**

During 5.1 (interquartile range: 3.1–5.6) years of follow-up, 21 ipsilateral cerebrovascular ischemic events were identified. Twelve new ipsilateral brain infarcts were identified on the 2-year neuro MRI. There was no association for IPH SIR or IPH volume with the clinical endpoint (hazard ratio (HR): 0.89 [95% confidence interval: 0.67–1.10] and HR: 0.91 [0.69–1.19] per 100-µL increase, respectively) nor with the imaging-based endpoint (odds ratio (OR): 1.04 [0.75–1.45] and OR: 1.21 [0.87–1.68] per 100-µL increase, respectively).

**Conclusion:**

IPH SIR and IPH volume were not associated with future ipsilateral ischemic cerebrovascular events. Therefore, quantitative assessment of IPH of SIR and volume does not seem to provide additional value beyond the presence of IPH for stroke risk assessment. Trial registration: The PARISK study was registered on ClinicalTrials.gov with ID NCT01208025 on September 21, 2010 (https://clinicaltrials.gov/study/NCT01208025).

## Graphical abstract

**Figure:**
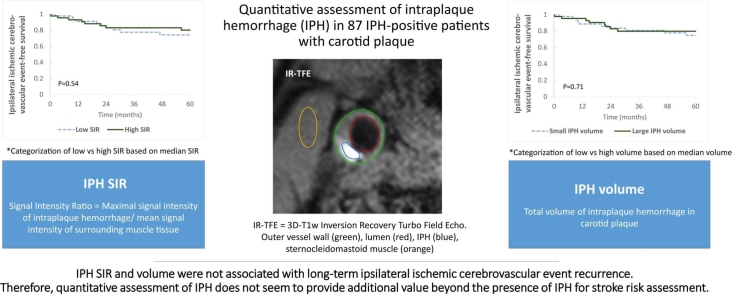

## 1 Background

Patients with carotid atherosclerotic plaque are at increased risk of (recurrent) ischemic cerebrovascular events. The main underlying cause of stroke in these patients is the rupture of a vulnerable carotid plaque, which can lead to thrombosis and embolization, and subsequently, ischemic stroke by the blockage of blood vessels further downstream. It has become apparent that besides the degree of stenosis, the composition of the carotid artery plaque largely determines the risk of stroke [1]. Key features that determine plaque vulnerability are a large lipid-rich necrotic core (LRNC), intraplaque hemorrhage (IPH), a thin or ruptured fibrous cap, and ulcerations [2]. These plaque features have repeatedly been shown to provide added information to the degree of stenosis with regard to the risk of stroke [1], [3], [4]. In particular, the presence of IPH, as identified with carotid cardiovascular magnetic resonance (CMR), is considered one of the major risk factors for stroke and has also been proposed to serve as an imaging biomarker for stroke risk assessment [5], [6].

Due to the superior soft tissue contrast compared to other imaging modalities, CMR has made the visualization of all the hallmarks of plaque vulnerability possible [2]. IPH is easy to identify as a hyperintense signal in the bulk of the plaque on hyper T_1_ weighted magnetic resonance (MR) images [2], [7]. It can be visualized using a dedicated CMR sequence that takes approximately 5 minutes with a standard neurovascular MRI coil [7], [8]. The MRI sequence Magnetization Prepared-RApid Gradient Echo, also known as inversion recovery turbo field echo (IR-TFE), is most commonly used for IPH detection and has been extensively validated [2].

It is possible to look beyond the presence of IPH and quantify IPH signal intensity and IPH volume. These measurements could prove to be interesting markers of plaque vulnerability since it was previously shown that the IPH signal intensity ratio (SIR) was significantly higher (5.8 ± 2.4 vs 4.7 ± 1.8, p = 0.004) on the symptomatic vs the asymptomatic side in a study of 31 patients with a recent ischemic cerebrovascular event and bilateral IPH [9]. The same study also reported a trend toward a higher IPH volume (150 ± 199 vs 88 ± 106 mm^3^) on the symptomatic vs the asymptomatic side. A larger IPH volume on computed tomography angiography (CTA) was also associated with cerebrovascular ischemic events (OR = 10.1, p = 0.03, 95% CI = 1.24–82.14) when comparing the IPH volume of 66 symptomatic and 102 asymptomatic plaques [10]. Considering the cross-sectional association that was reported between IPH SIR and volume and symptomatic plaques, we aimed to explore whether IPH SIR and volume are associated with future ischemic ipsilateral cerebrovascular events in symptomatic patients with mild-to-moderate carotid artery stenosis.

We hypothesize that an increase in IPH SIR or volume is associated with (1) a higher risk of recurrent ipsilateral stroke, transient ischemic attack (TIA), or amaurosis fugax, and (2) new ipsilateral infarcts on brain MRI.

## 2 Methods

### 2.1 Study population

To investigate the relationship between IPH signal intensity and volume in the carotid plaque and recurrent ipsilateral ischemic cerebrovascular events, patient data gathered during the Plaque At RISK (PARISK) study were used. Only patients with an IPH-positive status in the ipsilateral carotid plaque were used for the present analysis. The PARISK study was a prospective multicenter cohort study that included patients who were recently (<3 months) symptomatic (minor ischemic stroke, TIA, and amaurosis fugax) and had an ipsilateral carotid plaque of at least 2 mm, causing <70% stenosis (North American Symptomatic Carotid Endarterectomy Trial (NASCET) criteria) as determined by Doppler ultrasound or CTA. Patients with a probable cardiac source of embolism, clotting disorder, or severe comorbidity were excluded from study inclusion. Patients in the PARISK study had a clinical follow-up of 5 years. More details on the study design protocol have been described previously [11]. Institutional Review Board approval (Medisch Ethische Commissie azM/UM, approval number NL29116.068.09/MEC 09-2-082) was obtained and all patients gave written informed consent.

### 2.2 Magnetic resonance imaging

**Fig. 1:**
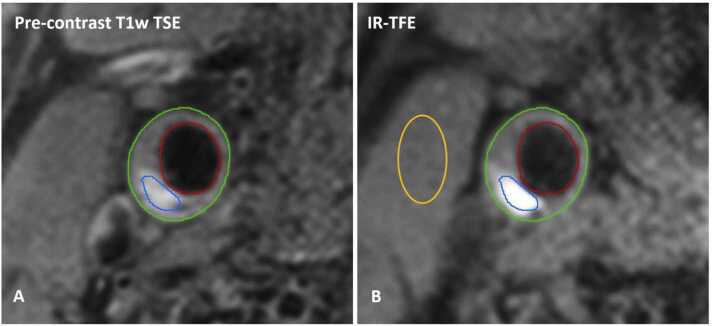
Carotid CMR images of an IPH-positive plaque. (A) The lumen (red) and outer vessel wall (green) were delineated on the pre-contrast T_1_w TSE image and used to determine the total wall volume. (B) Intraplaque hemorrhage (IPH) was delineated based on hyperintense signal in the bulk of the plaque on the 3D-T_1_w inversion recovery turbo field echo images. The IPH signal intensity ratio was defined as the maximum signal intensity within the IPH region (blue contour) divided by the average signal intensity of the adjacent muscle tissue (orange contour). *3D* three-dimensional, *IR-TFE* inversion recovery turbo field echo, *CMR* cardiovascular magnetic resonance, *T1w* T1-weighted, *TSE* turbo spin echo

The patients underwent brain MRI and carotid plaque CMR at baseline on a 3.0T MRI system (Discovery MR 750, GE Healthcare, Milwaukee, Wisconsin or Achieva, Philips, Best, the Netherlands) (Fig. 1). Details of the MRI protocol were previously described [11]. For carotid CMR, a dedicated eight-channel phased-array coil (Shanghai Chenguang Medical Technologies Co., Shanghai, China) was used for imaging of the carotid arteries in three centers; a dedicated four-channel carotid phased-array coil with an angulated setup (Machnet B.V., Roden, the Netherlands) was used in one center. After a scout scan, the following carotid CMR imaging sequences were acquired: three-dimensional (3D) Fast Spoiled Gradient Echo (FSPGR) and pre- and post-contrast 2D-T_1_w Double Inversion Recovery Fast Spin Echo (GE Healthcare) or 3D-T_1_w IR-TFE and pre- and post-contrast 2D-T_1_w Quadruple Inversion Recovery Turbo Spin Echo (TSE) (Philips). Fifteen transverse adjoining slices of 2 mm of the extracranial carotid artery, centered on the carotid plaque, were acquired. The neuro MRI examination was performed using a dedicated head coil, and this examination was repeated after 2 years.

### 2.3 Image analysis

The images were analyzed using dedicated vessel wall analysis software (VesselMASS; Leiden University Medical Center, Leiden, the Netherlands) by trained observers blinded to the clinical data as described previously [12]. Trained observers delineated the inner and outer vessel walls on the pre-contrast black blood T_1_w TSE MR images. Using CMR, IPH can be identified with a sensitivity of 87% (95% confidence interval (CI): 81–91%) and specificity of 92% (95% CI: 87–95%) [13]. IPH was delineated as a hyperintense region in the bulk of the carotid plaque compared to the adjacent muscle tissue on T_1_w-IR-TFE or FSPGR images. For each of the 15 slices, IPH SIR was calculated by dividing the maximum signal intensity of the voxels within the region of interest (ROI) identified as IPH, by the mean signal intensity within the manually drawn ROI within the adjacent muscle on the same slice, similar to previously reported methodology by an independent research group [14]. The maximum IPH SIR was then determined by selecting the highest SIR out of the 15 slices. The total plaque volume and IPH volume were automatically calculated based on the manually drawn contours and slice thickness. There was a good-to-excellent agreement between two trained observers that independently delineated IPH and drew an ROI in the muscle tissue. The inter-observer agreement on the quantification of the IPH volume was good (intraclass correlation coefficient (ICC): 0.78; 95% CI: 0.62–0.87). For IPH SIR, the inter-observer agreement was excellent (ICC: 0.98; 95% CI: 0.92–0.99). The percentage of IPH volume relative to total plaque volume was calculated by dividing IPH volume by total plaque volume. For an explorative sub-analysis, the lipid-rich necrotic core volume and the presence of a thin or ruptured fibrous cap were also assessed on MR images as previously described [15], [16], while ulcerations were identified on CTA scans as previously reported [17].

### 2.4 Clinical and imaging endpoints

For our primary analysis, clinical endpoints consisting of recurrent ipsilateral ischemic cerebrovascular events (stroke, TIA, and amaurosis fugax) were registered during follow-up after 3 months and thereafter yearly for up to 5 years based on patient interviews by a medical doctor using structured case record forms or by contacting the patient’s general practitioner (GP) in case the patient could not be reached. Minor stroke was defined as an episode of focal cerebral dysfunction of vascular origin, lasting for more than 24 hours or a non‐disabling stroke with a modified Rankin score ≤3. TIA was defined as an episode of temporary and focal cerebral dysfunction of vascular origin, lasting for a maximum of 24 hours, and leaving no persistent neurological deficit. We defined amaurosis fugax, also known as transient monocular blindness, as momentary visual loss occurring as a result of transient retinal ischemia of the carotid artery territory. Ischemic cerebrovascular events were verified by medical records of the hospital or GP and evaluated independently by two experienced vascular neurologists, blinded to the carotid MRI results. For our secondary analysis, imaging endpoints were registered and identified as new ipsilateral ischemic brain lesions on the brain MRI examination after 2 years. Therefore, it was possible to compare the baseline and follow-up MRIs to identify new cortical and subcortical infarcts. The brain MRI examinations were evaluated by an experienced neuroradiologist who was blinded to the clinical and carotid CMR data.

### 2.5 Statistical analysis

Baseline data are presented as the mean ± standard deviation (SD) when normally distributed, median with interquartile range (IQR) for non-normal data, or count (%) for categorical data. Normality was checked using a Kolmogorov–Smirnov test. Missing data on the co-variables were imputed using five iterations of multiple imputations. All statistical analyses were performed using SPSS statistical software (IBM SPSS Statistics 26; Chicago, Illinois) with p values <0.05 considered statistically significant.

### 2.6 Relation between IPH SIR and IPH volume and clinical endpoints

For the primary analysis, Cox proportional hazard models were used to investigate the association of IPH signal intensity, IPH volume, and IPH volume percentage of total vessel volume with the risk of recurrent ischemic stroke, TIA, or amaurosis fugax. We constructed three models: model 1: unadjusted, model 2: adjusted for age and sex, and model 3: adjusted for age, sex, and the degree of stenosis based on NASCET criteria. Hazard ratios (HRs) with corresponding 95% CIs for the Cox proportional hazards models are provided.

To further explore the relationship between IPH SIR, IPH volume, and clinical endpoints, the patients were split into two groups with a low vs high SIR or low vs high IPH volume based on the median SIR or median IPH volume of the population. Patients with a higher SIR or volume than the median of the population were categorized as having high IPH SIR or high IPH volume, respectively. Kaplan-Meier plots of cerebrovascular event-free survival in the low and high IPH SIR and IPH volume groups were created and assessed with a log-rank (Mantel-Cox) test.

### 2.7 Relation between IPH SIR and volume and imaging endpoints

As a secondary analysis, a chi-square test with Yates’s Continuity Correction was used to examine differences in median IPH SIR and IPH volume in patients with and without a new infarct on brain MRI. Logistic regression was used to determine the association of IPH signal intensity and IPH volume with new ipsilateral infarcts on neuro MRI. We constructed three models adjusted for the same factors as described above. For the logistic regression models, odds ratios (ORs) with corresponding 95% CIs are presented.

### 2.8 Explorative sub-analyses

A number of additional explorative sub-analyses were performed in this study. Given the potential diagnostic difficulty of identifying individuals with amaurosis fugax, we repeated the above-mentioned analyses while excluding patients who experienced amaurosis fugax at baseline and with a clinical endpoint of ipsilateral ischemic stroke and TIA only. In the present study, one center used an MRI system of a different MRI manufacturer. Therefore, in another sub-analysis, the MRI manufacturer was included as a confounder. Last, we also performed an explorative analysis where we included a larger number of potential confounders: age, sex, degree of carotid stenosis, the classification of the index event (stroke, TIA, amaurosis fugax), hypertension, diabetes, statin use before the index event, lipid-rich necrotic core volume, the presence of a thin or ruptured fibrous cap, and plaque ulcerations.

## 3 Results

Baseline carotid MRI data were available for 224 patients. After the exclusion of IPH-negative patients (n = 137), 87 IPH-positive patients were included in the present study.

The median age of these patients was 71 (IQR: 66–75) years and 87% (76/87) of the patients were male. The baseline clinical and imaging characteristics of the patients are provided in Table 1. The median IPH SIR was 2.6 (IQR: 2.0–4.5) and the median IPH volume was 115 µL (IQR: 29–238) in the total population.

**Table 1:** Clinical and imaging characteristics at baseline.

*Clinical characteristics at baseline (n* *=* *87)*	
Age (in years)	71 (66–75)
Male sex	87% (76/87)
Caucasian	94% (78/83)
Current smoking	14% (12/87)
BMI, kg/m^3^	25.5 (24.1–27.8)
Hypertension	81% (64/79)
Hypercholesterolemia	79% (63/80)
Diabetes mellitus	22% (17/79)
History of ischemic heart disease	24% (21/87)
Use of statins	50% (43/86)
Use of antihypertensive drugs	66% (57/87)
Use of antithrombotics	52% (45/86)
Classification baseline event	
Cerebral stroke	53% (46/87)
Cerebral TIA	36% (31/87)
Amaurosis fugax	11% (10/87)

*Imaging parameters at baseline (n* *=* *87)*	
Degree of stenosis (NASCET)	23 (5–36)
Time between index event and CMR (in days)	51 (33–70)
IPH signal intensity ratio	2.6 (2.0–4.5)
IPH volume (in µL)	115 (29–238)
% IPH volume of total plaque volume	7.7 (2.0–16.3)
Lipid-rich necrotic core volume (in µL, excluding IPH volume)	65 (29–125)
Presence of a thin or ruptured fibrous cap	80% (67/84)
Presence of an ulceration	41% (30/73)

*BMI* body mass index*, IPH* intraplaque hemorrhage*, CMR* cardiovascular magnetic resonance *, NASCET* North American Symptomatic Carotid Endarterectomy Trial, *TIA* transient ischemic attack*.*

Values are presented as the median (IQR) or n (%) of the non-imputed original values. The degree of stenosis was determined based on CTA measurements. All other imaging characteristics were MRI-based. Missing values included plaque ulcerations (n = 14), ethnicity (n = 4), thin or ruptured fibrous cap (n = 3), body mass index (n = 2), hypertension (n = 8), hypercholesterolemia (n = 7), diabetes mellitus (n = 8), statin use (n = 1), antithrombotic use (n = 1), and degree of stenosis (n = 13).

### 3.1 Relation between IPH SIR and IPH volume and clinical endpoints

During a median clinical follow-up of 5.1 (IQR: 3.1–5.6) years, 21 patients experienced a total of 22 clinical ipsilateral ischemic events (i.e., 9 ischemic strokes, 11 TIAs, and 2 amaurosis fugax events). Note that one patient of the listed event count suffered from a second clinical event during follow-up and had an ipsilateral TIA and an ipsilateral ischemic stroke, of which only the first event, in this case, the ipsilateral TIA, was used for survival analysis of the cerebrovascular event-free survival time.

**Fig. 2:**
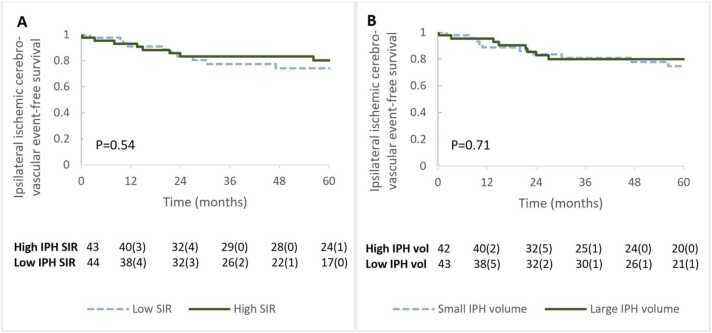
Clinical event-free survival in patients categorized based on IPH signal intensity ratio and IPH volume. Ipsilateral ischemic cerebrovascular event (stroke, TIA, amaurosis fugax)-free survival of patients was categorized into (A) low and high IPH signal intensity, (B) low and high IPH volume, based on the median of the population. IPH SIR above 2.6 and IPH volume above 115 µL were categorized as “High IPH SIR” and “High IPH volume,” respectively. Numbers at risk during each year are presented as the number at risk followed by (the number of patients with a new ipsilateral ischemic cerebrovascular event in the preceding year). *IPH* intraplaque hemorrhage, *SIR* signal intensity ratio, *TIA* transient ischemic attack, *vol* volume.

For illustrative purposes only, the cerebrovascular event-free survival probability of patients with a high vs low IPH SIR and high vs low IPH volume (Fig. 2) was visualized. In our primary analysis, the relationship between IPH SIR and recurrent clinical events was analyzed using Cox proportional hazard models (Table 2). No significant association (HR: 0.89 [95% CI: 0.71–1.12] per unit increase, p = 0.35) between IPH SIR and clinical events was observed. The HR did not change after adjusting for age and sex (HR: 0.87 [95% CI: 0.68–1.10] per unit increase, p = 0.25) or for age, sex, and the degree of stenosis (HR: 0.86 [95% CI: 0.67–1.10] per unit increase, p = 0.22). To provide a better indication of the potential strength of the association between IPH SIR and new cerebrovascular ischemic events, the HR for an increase in the IPH SIR by the value of the IQR (IQR = Q3–Q1) was calculated. With an IQR of 2.5, the HR was 0.66 (95% CI: 0.37–1.27) per 2.5 increase (or IQR) in IPH SIR.

**Table 2:** Cox proportional hazard and logistic regression models for clinical and imaging endpoints.

	IPH SIR		IPH volume
*Clinical endpoints (n* *=* *21)*			
Model 1	0.89 [0.71–1.12]	0.94 [0.73–1.21]	
Model 2	0.87 [0.68–1.10]	0.92 [0.71–1.20]	
Model 3	0.86 [0.67–1.10]	0.91 [0.69–1.19]	
*Imaging endpoints (n* *=* *12)*			
Model 1	1.01 [0.73–1.38]	1.13 [0.85–1.50]	
Model 2	1.00 [0.73–1.38]	1.11 [0.83–1.48]	
Model 3	1.04 [0.75–1.45]	1.21 [0.87–1.68]	

*IPH* intraplaque hemorrhage*, SIR* signal intensity ratio*.*

Also for IPH volume, there was no significant association between IPH volume and clinical endpoints in the Cox proportional hazards model (HR: 0.94 [95% CI: 0.73–1.21] per 100-µL increase, p = 0.63). After adjusting for age and sex (HR: 0.92 [95% CI: 0.71–1.20] per 100-µL increase, p = 0.54) or for age, sex, and the degree of stenosis (HR: 0.91 [95% CI: 0.69–1.19] per 100-µL increase, p = 0.48), no significant association between IPH volume and ipsilateral ischemic cerebrovascular events was observed. Considering that the IQR of the IPH volume was 209 µL, the HR of an increase in IPH volume by the value of the IQR was 0.82 (95% CI: 0.46–1.44) per 209-µL increase.

Additionally, we explored whether IPH volume as a percentage of the total vessel wall volume was associated with future clinical events of ischemic stroke, TIA, or amaurosis fugax. Similarly, for the percentage of IPH, no association with clinical endpoints was found in the unadjusted analysis (HR: 0.19 [95% CI: 0.00–28.76], p = 0.52), the analysis adjusted for age and sex (HR: 0.12 [95% CI: 0.00–19.97], p = 0.42), or the analysis adjusted for age, sex, and the degree of stenosis (HR: 0.08 [95% CI: 0.00–17.71], p = 0.36).

The results are presented as the HR for clinical endpoints or OR for imaging endpoints including 95% CI. HRs and ORs of IPH volume are per 100-µL increase in volume. Model 1 presents the unadjusted analysis, model 2 is adjusted for age and sex, and model 3 is adjusted for age, sex, and the degree of carotid stenosis.

### 3.2 Relation between IPH SIR and volume and imaging endpoints

At the 2-year follow-up, 69 of the 87 patients who had an IPH-positive ipsilateral carotid plaque underwent a brain MRI to identify any new (silent) ipsilateral infarcts. Out of the 18 patients who did not undergo a follow-up brain MRI, 7 patients refused the examination, 6 patients were lost to follow-up, 2 patients died, 2 brain MRIs had an incomplete protocol due to technical difficulties, and for 1 patient no baseline brain MRI was available. Out of 69 patients with a follow-up brain MRI, 12 patients had a new ipsilateral brain infarct as identified on brain MRI. The median SIR of IPH did not significantly differ (p = 0.71) between patients who had a new ipsilateral (silent) infarct during follow-up and patients who had not (2.9 [IQR: 2.0–5.1] vs 3.6 [IQR: 2.1–4.8], respectively). The median IPH volume in patients who had a new ipsilateral (silent) infarct was elevated, although not significantly higher, compared to patients without a new ipsilateral (silent) infarct on brain MRI (113.6 [IQR: 28.8–229.8] vs 167.1 [IQR: 48.1–344.4], p = 0.31).

In our secondary analysis for new ipsilateral infarcts after 2 years of follow-up, using unadjusted logistic regression, no significant association between IPH SIR and the imaging endpoint on neuro MRI was observed (OR: 1.01 [95% CI: 0.73–1.38], p = 0.96). After adjusting for age and sex (OR: 1.00 [95% CI: 0.73–1.38], p = 0.99) or for age, sex, and the degree of stenosis (OR: 1.04 [95% CI: 0.75–1.45], p = 0.82), the results did not change markedly. The OR of having a new ipsilateral infarct was 1.10 (95% CI: 0.49–2.53) per 2.5 increase in IPH SIR (the value of the IQR). Also for IPH volume, the unadjusted results showed no relationship with the imaging endpoint (OR: 1.13 [95% CI: 0.85–1.50] per 100-µL increase, p = 0.40) which remained non-significant after correction for age and sex (OR: 1.11 [95% CI: 0.83–1.48] per 100-µL increase, p = 0.47) or age, sex, and the degree of stenosis (OR: 1.21 [95% CI: 0.87–1.68] per 100-µL increase, p = 0.25). For an increase in IPH volume by 209 µL (the value of the IQR), the OR for the risk of a new ipsilateral infarct on brain MRI is 1.49 (95% CI: 0.75–2.96) per 209-µL increase.

### 3.3 Explorative sub-analyses

After the exclusion of patients with amaurosis fugax at baseline and after exclusion of amaurosis fugax from the clinical endpoint, a dataset of 77 patients remained with 16 clinical endpoints (TIA or stroke) during follow-up. The exclusion of amaurosis fugax resulted in similar findings with an adjusted HR for IPH SIR and IPH volume of 0.86 [95% CI: 0.64–1.16] per unit increase and 0.89 [95% CI: 0.65–1.22] per 100-µL increase. For 62 of those patients, the status of the imaging endpoint was known, resulting in 11 imaging endpoints. For the imaging endpoint, the findings also remained similar after the exclusion of amaurosis fugax with an adjusted OR of 0.98 [95% CI: 0.68–1.41] per unit increase and 1.15 [95% CI: 0.82–1.61] per 100-µL increase for IPH SIR and IPH volume, respectively.

The additional explorative analysis where we corrected for more confounders, i.e., age, sex, the degree of carotid stenosis, the classification of the index event (stroke, TIA, or amaurosis fugax), hypertension, diabetes, statin use before the index event, lipid-rich necrotic core volume, the presence of a thin or ruptured fibrous cap, and plaque ulcerations, also resulted in similar HRs and ORs. IPH SIR had an adjusted HR of 0.85 [95% CI: 0.62–1.17] for new clinical events and 1.04 [95% CI: 0.72–1.49] for the imaging endpoint. IPH volume per 100-µL increase had an adjusted HR of 0.89 [95% CI: 0.65–1.22] for new clinical events and an adjusted OR of 1.16 [95% CI: 0.84–1.61] for the imaging endpoint.

Moreover, similar results were observed when adding the MRI manufacturer as an additional confounder in an explorative analysis (Supplementary Table 1).

## 4 Discussion

In this study, a high IPH SIR or a large IPH volume were not associated with recurrent ipsilateral ischemic cerebrovascular events or new ipsilateral infarct on brain MRI. Hence, IPH SIR and IPH volume provide no additional information with regard to the risk of stroke beyond the mere presence of IPH.

A few cross-sectional studies have reported an association between IPH signal intensity, IPH volume, and ipsilateral ischemic events. Wang et al. [9] reported a higher IPH SIR (5.8 ± 2.4 vs 4.7 ± 1.8, p = 0.004) in symptomatic versus asymptomatic plaques; however, due to the large SD, the feasibility of clinical implementation as a diagnostic factor is limited. Liu et al. [18] reported IPH volume to be significantly associated with acute ipsilateral cerebral infarcts, also after adjusting for plaque and LRNC volume (OR = 4.044, p = 0.024). However, it should be noted that out of the 687 recruited patients in the study by Liu et al., only 17.2% of patients were IPH-positive and patients without IPH were not excluded from the analysis. Therefore, the reported association may have been affected by the mere presence of IPH rather than IPH volume. In a smaller study with 85 patients of whom 70 were symptomatic and 15 were asymptomatic, only IPH-positive carotid arteries were included resulting in the finding of no association between IPH SIR and ipsilateral ischemic events [14]. All these studies had a cross-sectional study design, while at present we performed a longitudinal study that enabled us to study the association with the risk of future ipsilateral ischemic events.

In our study, no association was found between IPH SIR and the risk of ipsilateral ischemic event recurrence. Since high IPH SIR is assumed to be related to a fresh IPH plaque, which may lead to an increase in mean wall thickness and a decrease in lumen volume, the current findings were not anticipated [19]. Large fluctuations in IPH SIR are reported in symptomatic carotid arteries, which may diminish the magnitude of a potential association of the parameter to event recurrence [19]. In our explorative study, we were interested in examining whether there is an association between IPH SIR or volume and any ipsilateral ischemic events occurring during a long-term follow-up of 5 years. To our knowledge, there have been no previous studies reporting on the association of IPH SIR and IPH volume for identifying who will be at risk for a recurrent ipsilateral cerebrovascular event.

In contrast to IPH SIR and volume, IPH presence itself showed a strong and independent association with cerebrovascular event recurrence as previously reported by the PARISK study and a large meta-analysis [5], [6]. While quantification of IPH SIR and volume unfortunately does not offer improved prognostic value for the risk of recurrent ipsilateral cerebrovascular events, the finding is important for clinical practice since the delineation of IPH is time-consuming. The present study shows that this does not provide added value for risk stratification. The identification of IPH presence on MRI as a predictor is much easier in daily clinical practice compared to the additional quantification of IPH signal intensity and volume. The quantitative analysis of IPH signal intensity and volume would require additional training and increased image analysis time compared to only assessing IPH presence.

## 5 Limitations

The major limitation of our study is the small sample size, which was powered to correct for only three confounders. Still, the HRs for IPH SIR and IPH volume reported in the present study, even at the upper bound of the 95% CI, are small and, therefore, not clinically relevant. In two additional explorative sub-analyses, we corrected for an extended set of clinical and imaging variables and for confounding by the manufacturer of the MRI system. Note that we were unable to correct for the National Institutes of Health Stroke Scale score, since this variable was not determined in the PARISK study, but only patients with a TIA or minor stroke with a modified Rankin score of ≤3 were eligible for inclusion. All presented sub-analyses showed similar results as the primary analysis, which is an indication of the robustness of our findings. A post-hoc power calculation with 80% power and an alpha of 0.05 was performed using the “ssizeEpiCont” function of the package “powerSurvEpi” (Qiu W, et al (2021); version 0.1.3) in R Statistical Software (version 4.3.0; R Core Team 2021). For our primary analysis, the current study was appropriately powered to detect HRs <0.72 and >1.39 for a unit increase in IPH SIR and to detect an HR <0.70 and >1.43 for a 100-µL increase in IPH volume. For the explorative sub-analysis with 10 confounders, the study was powered to detect HRs <0.69 and >1.45 for a unit increase in IPH SIR, while for IPH volume HRs <0.67 and >1.50 could be detected per 100-µL increase. Given the detectable effect range, sample size improvement could indicate at best a weak association with new ipsilateral ischemic events.

Second, our primary analysis included amaurosis fugax and TIA as a clinical endpoint. Given that amaurosis fugax may be more challenging to diagnose, we also performed a sub-analysis excluding patients with amaurosis fugax at baseline and excluding amaurosis fugax from the clinical endpoint, which led to similar findings as the primary analysis. A separate analysis for an ipsilateral ischemic stroke-only clinical endpoint could be of further interest; however, the stroke incidence was insufficient in the PARISK study to offer reliable effect estimations. With regards to diagnostic accuracy, all diagnoses were verified by medical records of the hospital and/or the GP and evaluated independently by two experienced vascular neurologists. We agree that TIA and amaurosis fugax diagnosis is generally less reliable than stroke diagnosis; however, by independent evaluation of experts, we have minimized errors in diagnosis.

Third, we focused on the ipsilateral side, since revascularization is usually considered for the symptomatic artery and, therefore, it is important to investigate the association between symptomatic plaque characteristics and the risk for cerebral ischemia. We did not assess the IPH SIR and volume in the asymptomatic, contralateral, carotid artery. This should be investigated in future studies.

Last, a cardio-embolic or intracranial atherosclerotic plaque cause of stroke cannot be excluded from our study. However, the baseline inclusion and exclusion criteria of the PARISK study and the independent verification of clinical events by two experienced vascular neurologists reduced the risk of reporting stroke recurrence by other causes than the extracranial carotid artery territory.

## 6 Conclusions

In our explorative analysis, IPH SIR and volume were not associated with the risk of ipsilateral ischemic cerebrovascular events. In contrast, we previously reported a strong predictive value for stroke risk for the presence of IPH on MRI in the PARISK study. Therefore, simply identifying the presence of IPH seems to be sufficient.

## Funding

This research was supported by the Netherlands Heart Foundation and performed within the framework of the Centre for Translational Molecular Medicine (www.ctmm.nl), project PARISK (Plaque At RISK; grant number 01C-202). Prof. M.E. Kooi is supported by an Aspasia Grant 2018/SGw/00460457 from NWO.

Author contributions

**Rob van der Geest:** Writing – review and editing, Software. **Paul Nederkoorn:** Writing – review and editing, Investigation, Funding acquisition, Data curation, Conceptualization. **Jeroen Hendrikse:** Writing – review and editing, Investigation, Funding acquisition, Data curation, Conceptualization. **Daniel Bos:** Writing – review and editing. **Alida A. Postma:** Writing – review and editing. **Narender P. van Orshoven:** Writing – review and editing, Data curation. **Tobien A.H.C.M.L. Schreuder:** Writing – review and editing, Data curation. **Timothy C.D. Goring:** Writing – review and editing. **M. Eline Kooi:** Writing – review and editing, Supervision, Methodology, Funding acquisition, Conceptualization. **Dianne H.K. van Dam-Nolen:** Writing – review and editing. **Werner H. Mess:** Writing – review and editing, Funding acquisition, Conceptualization. **Mueez Aizaz:** Writing – review and editing, Methodology, Formal analysis. **Robert J. van Oostenbrugge:** Writing – review and editing, Conceptualization. **Kelly P.H. Nies:** Writing – original draft, Methodology, Formal analysis, Data curation.

## Ethics approval and consent

Institutional Review Board approval (Medisch Ethische Commissie azM/UM, approval number NL29116.068.09/MEC 09-2-082) was obtained and all patients gave written informed consent.

## Declaration of competing interests

The authors declare that they have no known competing financial interests or personal relationships that could have appeared to influence the work reported in this paper.
