# Supplementary material for: Signal intensity and volume of carotid intraplaque hemorrhage on magnetic resonance imaging and the risk of ipsilateral cerebrovascular events: The Plaque At RISK (PARISK) study
Source: J Cardiovasc Magn Reson. 2024 Jun 13;26(2):101049. doi: 10.1016/j.jocmr.2024.101049 (PMC11282977; doi:10.1016/j.jocmr.2024.101049)
Supplement: Supplementary file 1 — Supplementary material [file mmc1.docx]

## **Supplemental Table 1: Cox proportional hazard and logistic regression models for clinical and imaging endpoints for the explorative sub-analysis with the manufacturer of the MRI system as additional confounder**

In the Plaque At Risk (PARISK) study MRI systems from two different MRI manufacturers (GE Healthcare and Philips) were used. In an additional explorative analysis, we have added the manufacturer of the MRI system as an additional confounder to our model. It can be observed that these findings are very similar to the main analysis without adding the manufacturer of the MRI system as confounder.

|  | **IPH SIR** | | **IPH volume** |
| --- | --- | --- | --- |
| **Clinical endpoints (n=21)** | | | |
| Model 1 | 0.91 [0.70-1.19] | 0.94 [0.73-1.21] | |
| Model 2 | 0.88 [0.67-1.16] | 0.92 [0.70-1.20] | |
| Model 3 | 0.87 [0.65-1.16] | 0.91 [0.69-1.20] | |
| **Imaging endpoints (n=12)** | | | |
| Model 1 | 1.00 [0.70-1.42] | 1.13 [0.85-1.50] | |
| Model 2 | 0.99 [0.69-1.41] | 1.11 [0.83-1.48] | |
| Model 3 | 1.04 [0.71-1.51] | 1.21 [0.87-1.67] | |

The results are presented as the hazard ratio (HR) for clinical endpoints or odds ratio (OR) for imaging endpoints including 95% confidence interval. HRs and ORs of intraplaque hemorrhage (IPH) volume are per 100 µl increase in volume. Model 1 is adjusted for the manufacturer of the MRI system only. Model 2 is adjusted for age , sex and manufacturer of the MRI system, and Model 3 is adjusted for age, sex, the degree of carotid stenosis and the manufacturer of the MRI system.
